# Translating Geroscience Into Clinical Longevity Dermatology: From Mechanisms of Aging to Skin‐Centered Interventions

**DOI:** 10.1111/jocd.70616

**Published:** 2025-12-24

**Authors:** Diala Haykal

**Affiliations:** ^1^ Centre Médical Laser Palaiseau Private Practice Palaiseau France

**Keywords:** biological markers, cosmetic dermatology, geroscience, longevity, precision medicine, skin aging

## Abstract

**Background:**

Longevity medicine is an emerging clinical framework aimed at extending healthspan by targeting the biological mechanisms of aging rather than treating disease in isolation. Geroscience, which investigates the molecular and cellular pathways linking aging to chronic pathology, provides the scientific foundation for this approach. Dermatology is uniquely positioned within this paradigm, as the skin represents both a visible marker of biological aging and an accessible source of biomarkers.

**Objective:**

To explore how principles of geroscience can be translated into clinical dermatology and cosmetic practice, with a focus on skin‐centered biomarkers, artificial intelligence (AI), and preventive longevity‐oriented interventions.

**Methods:**

This piece integrates current evidence from geroscience, dermatologic aging research, microbiome science, and AI‐driven analytics to examine emerging models of longevity‐focused dermatologic care. Conceptual frameworks, clinical readiness of interventions, and ethical considerations are critically discussed.

**Results:**

Advances in biological aging biomarkers, including epigenetic clocks, inflammatory signatures, mitochondrial and metabolic markers, and skin microbiome profiling, offer promising tools for assessing cutaneous and systemic aging. AI‐enabled platforms facilitate the integration of multidimensional data, enabling refined biological age assessment and potential prediction of treatment responses. However, most longevity‐oriented diagnostics and interventions remain in early or experimental stages, requiring rigorous validation before routine clinical adoption.

**Conclusion:**

Dermatology can serve as a translational bridge between geroscience and clinical longevity medicine by integrating validated skin biomarkers, aesthetic procedures, and preventive strategies within an evidence‐based framework. Careful attention to scientific limitations, ethical considerations, and health equity is essential to ensure responsible implementation. Dermatologists would play a key role in shaping clinically sound, prevention‐focused longevity care centered on long‐term skin health and resilience.

## 1 Introduction

Medicine is undergoing a gradual shift from reactive treatment toward preventive and personalized approaches. Within this context, longevity medicine has emerged as a conceptual and translational bridge between basic aging science and clinical practice [1]. Geroscience refers to the study of biological mechanisms that link aging to disease, while longevity medicine denotes the clinical application of these principles to extend healthspan. The term precision geromedicine is used to describe data‐driven personalization within this field, integrating biomarkers and artificial intelligence (AI) to tailor interventions to individual biological profiles.

This commentary reflects on how the principles of geroscience can be meaningfully integrated into dermatology, a field that uniquely combines visible manifestations of aging with measurable biological indicators. It aims to offer a critical and forward‐looking perspective on emerging biomarkers, AI and clinical models that may guide responsible implementation of longevity‐oriented dermatologic care, while acknowledging current scientific, ethical, and regulatory limitations [2].

### 1.1 From Biology to Clinic: The Core of Longevity Medicine

The clinical value of geroscience lies in its ability to stratify patients according to biological rather than chronological age. Epigenetic clocks, proteomic signatures, and microbiome shifts have shown potential to capture aspects of aging biology that correlate with morbidity and mortality. Yet these measures are still evolving: reproducibility, standardization, and predictive accuracy remain active areas of debate. Clinical translation should therefore be understood less as immediate application and more as hypothesis‐driven integration. For example, identifying a patient with accelerated skin aging may encourage closer monitoring, lifestyle modification, or inclusion in preventive trials. Cosmetic dermatology can integrate these tools by combining traditional aesthetic endpoints with systemic biomarkers. Such approaches are promising, but they remain far from demonstrating that clinicians can reliably prevent age‐related diseases at scale [3, 4].

### 1.2 Biomarkers and Clinical Relevance

Biomarkers such as DNA methylation clocks (Horvath, GrimAge, PhenoAge), markers of chronic inflammation (“inflammaging”), senescence‐associated secretory phenotype (SASP) factors, NAD^+^ metabolism, and mitochondrial function offer multidimensional insights into biological aging. Functional proxies, frailty indices, grip strength, and gait speed remain practical indicators of physiological reserve [5]. While these biomarkers are increasingly used in research and pilot clinical programs, their role in routine medicine is not yet established. The value of such measures will depend on whether they can predict intervention outcomes, stratify trial populations, and serve as surrogate endpoints. Without rigorous validation, they risk being misappropriated in commercial “anti‐aging” markets [6].

Metabolic and mitochondrial function is assessed through markers such as NAD^+^/NADH ratios, lactate levels, and mitochondrial DNA copy number, offering insight into cellular energy balance and oxidative stress. The emergence of proteomic and transcriptomic profiling adds another layer, enabling the detection of age‐related shifts in gene and protein expression linked to pathways such as autophagy, DNA repair, and metabolic regulation [2, 7].

Another important domain is the human microbiome. Alterations in the composition and diversity of gut and skin microbiota have been associated with systemic aging processes, including immune modulation, metabolic efficiency, and cognitive function. Microbiome‐derived metabolites such as short‐chain fatty acids and trimethylamine N‐oxide are gaining traction as biomarkers of both local and systemic health [8].

While still developing, biological aging markers offer valuable insight into cutaneous aging processes. DNA methylation clocks, mitochondrial signatures, and proteomic profiles each capture complementary aspects of biological age. However, their performance can vary depending on tissue type, environmental exposure, and analytic methods. Ongoing efforts to standardize assays and validate them against skin‐specific endpoints are promising steps toward improving reproducibility and clinical utility. As this evidence base expands, these biomarkers are likely to evolve from exploratory research tools into meaningful indicators of skin health and biological aging.

### 1.3 Artificial Intelligence as a Catalyst

AI offers a powerful means to analyze complex, high‐dimensional aging datasets. By processing complex, multi‐layered datasets that are beyond human cognitive capacity, AI can reveal hidden patterns in gene expression, metabolic regulation, immune aging, and stress response. Machine learning algorithms are used to identify predictive biomarkers, segment patient populations by aging phenotype, and simulate the impact of various interventions on long‐term health outcomes. AI also plays a growing role in the development of digital twins, virtual models of patients that integrate real‐time physiological data with predictive modeling to inform clinical decisions [7, 9].

The use of AI in longevity medicine is not limited to research environments. In clinics, AI‐enabled tools are already being used to interpret epigenetic clocks, guide decisions around lifestyle modification, and forecast response to specific therapies such as senolytics or NAD+ boosters. These technologies support physicians in delivering more precise, data‐informed care that adapts dynamically to the evolving biological state of the patient [10, 11, 12].

### 1.4 Clinical Implementation: A Dermatology Model of Longevity Care

Integrating geroscience into cosmetic dermatology requires reorientation of traditional workflows. Physicians must not only master aesthetic techniques but also develop proficiency in the biology of aging, biomarker interpretation, and the use of AI in clinical decision‐making. This shift demands professional education through longevity‐focused programs and continuous engagement with emerging research. Table 1 provides an overview of longevity‐oriented interventions categorized by their degree of clinical readiness, reflecting the continuum from validated dermatologic strategies to experimental frontiers. Once this foundation is established, clinicians can begin to incorporate biological age assessments and resilience indicators into their patient evaluations, thereby moving beyond chronological age as the sole determinant of care [13].

**TABLE 1:** Longevity‐oriented interventions classified by clinical readiness.

Readiness level	Examples	Clinical status/notes
Clinically established	Lifestyle optimization, nutrition, sleep regulation, photoprotection, topical formulations, energy‐based rejuvenation devices (lasers, RF, ultrasound)	Supported by dermatologic evidence; routinely applied in clinical settings.
Emerging	Senotherapeutics (senolytic peptides, rapalogs), NAD^+^ modulation, microbiome‐targeted interventions, AI‐assisted diagnostics	Under early‐phase clinical exploration; promising but not standardized.
Experimental	Exosome‐based therapies, stem‐cell infusions, gene‐editing or reprogramming approaches	Conceptual or preclinical; lacking validated dermatologic or systemic longevity outcomes.

Longevity‐focused dermatology also necessitates a multidisciplinary model that brings together expertise from nutrition, exercise physiology, sleep medicine, regenerative medicine, and psychological well‐being. Unlike the traditional episodic nature of dermatologic consultations, longevity care emphasizes longitudinal relationships in which patients are monitored over time for improvements or deviations in their biological profile. Wearable technologies, home‐based diagnostics, and mobile health applications are expected to play a critical role in enabling this model, allowing continuous data collection that can guide early interventions [14].

**FIGURE 1:**
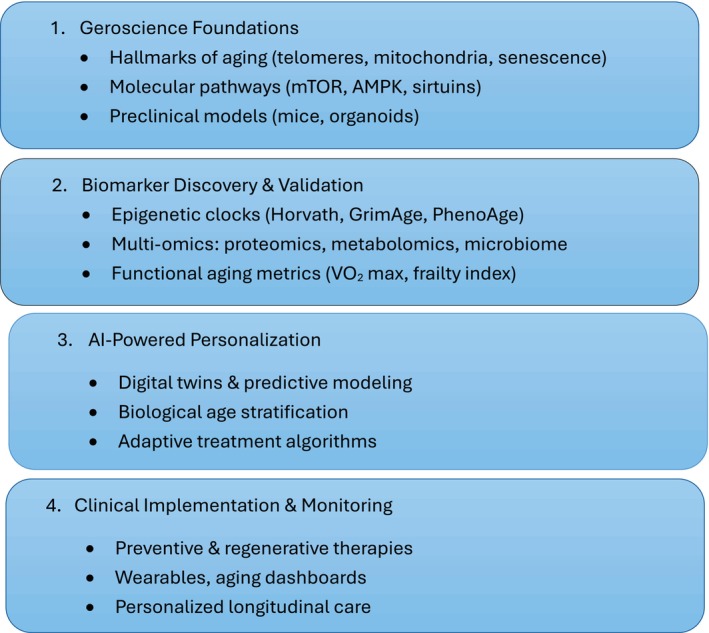
Translational framework of longevity medicine: from geroscience to application.

Therapeutic strategies should be viewed within a translational continuum. Currently, lifestyle modification, nutrition, sleep regulation, and topical or device‐based rejuvenation are the only clinically validated interventions for cutaneous aging. Agents such as senotherapeutics, NAD^+^ boosters, or microbiome modulators remain in early‐phase exploration. Stem cell infusions and exosome therapies, while conceptually promising, are experimental and lack validated dermatologic or systemic outcomes (Figure 1) [15, 16].

### 1.5 Ethical, Regulatory, and Equity Considerations

The translation of aging science into dermatology raises significant ethical and regulatory challenges. Many diagnostics and interventions in longevity medicine remain outside traditional reimbursement frameworks, which risks restricting access to patients who can afford out‐of‐pocket care and widening disparities in healthcare. Simultaneously, the integration of AI into clinical practice introduces questions of transparency, accountability, and bias. Ensuring that AI‐driven decision‐making remains auditable and equitable is essential for patient trust. Informed consent processes must also evolve to reflect the novel nature of biological age testing, continuous monitoring, and long‐term risk prediction [17].

In dermatology, informed consent must evolve to address the emerging realities of biological age testing, long‐term data monitoring, and predictive analytics. Consent discussions should clearly outline the off‐label or experimental nature of longevity‐oriented interventions, including senolytic injectables and exosome‐based topicals [18]. Moreover, AI‐driven skin age estimators and diagnostic tools require rigorous validation across diverse skin phototypes, as algorithms trained predominantly on lighter tones risk perpetuating bias and diagnostic inaccuracy. Transparent calibration across the full Fitzpatrick spectrum is therefore essential to ensure fairness and safety.

Regulatory authorities are beginning to explore frameworks to support longevity‐focused care. As biological age gains recognition as a clinical endpoint and as therapies targeting aging advance toward mainstream adoption, internationally harmonized guidelines will be vital to standardize implementation and safeguard patient welfare [19].

### 1.6 The Future of Geroscience‐Driven Clinical Practice

The fusion of geroscience, longevity medicine, and AI is laying the foundation for a healthcare paradigm in which aging is modifiable, resilience is measurable, and preventive care is highly personalized. Clinics that adopt these principles are not simply treating disease; they are promoting vitality, cognitive function, and independence across the lifespan [20]. In the near future, it is conceivable that longevity clinics will incorporate AI‐enhanced diagnostic systems, individualized aging dashboards, and continuously updated therapeutic libraries informed by real‐world evidence. Such an evolution would not only benefit individual patients but also help reduce the broader societal and economic burden of age‐related diseases [13, 21].

### 1.7 Diversity and Inclusivity

Aging phenotypes vary substantially according to sex, ethnicity, and genetic background, shaping both the biological mechanisms of skin aging and the accuracy of digital assessment tools. Differences in melanin density, dermal thickness, collagen cross‐linking, and inflammatory response contribute to distinct aging trajectories across populations. These variations influence not only the presentation of wrinkles, pigmentation, and skin laxity but also the efficacy and safety profiles of energy‐based devices and injectables.

AI models trained on non‐representative datasets may misinterpret these phenotypic variations, leading to biased predictions of “skin age” or inappropriate treatment recommendations. Studies have shown that diversity in datasets significantly enhances the accuracy and fairness of AI‐based age prediction and diagnostic models [22, 23]. Ensuring balanced inclusion of all Fitzpatrick phototypes, genders, and age groups is therefore essential to avoid algorithmic bias and to enable equitable dermatologic care.

Beyond biology, perceptions of youthfulness and aging differ across cultures, influenced by societal norms, aesthetic ideals, and environmental exposure. Incorporating global representation in longevity research and recognizing these cultural dimensions enrich both the scientific and ethical foundations of dermatology's engagement with longevity medicine.

## 2 Conclusion

Longevity medicine presents an ambitious vision for applying geroscience insights within clinical settings, yet the field remains in its formative stages. Current biomarkers and AI‐driven tools offer promising approaches to assessing biological age and physiological resilience, but no intervention has yet demonstrated the ability to prevent or reverse major age‐related diseases such as cancer, Alzheimer's disease, or osteoarthritis. To avoid repeating the unfulfilled promises of past “anti‐aging” movements, it is essential to approach longevity medicine through a critical, evidence‐based perspective. This means acknowledging current limitations, prioritizing well‐designed clinical studies, and addressing the ethical and regulatory considerations that will shape its safe and equitable integration into dermatologic and aesthetic practice.

## Funding

The author has nothing to report.

## Ethics Statement

The author has nothing to report.

## Conflicts of Interest

The author declares no conflicts of interest.

## Data Availability

The author has nothing to report.
